# Development of a Blockchain-Based Platform to Enable Indigenous Data Sovereignty and Shared Research Participation With Indigenous Communities: Technology Prototyping and Community Engagement Study

**DOI:** 10.2196/90247

**Published:** 2026-07-28

**Authors:** Tim Mackey, Alec J Calac, Tiana J McMann, Ken Miyachi, Samm Hurst, Keolu Fox, Burt Dillabaugh, Kelle Dhein, Guthrie Ducheneaux, Joe Yracheta

**Affiliations:** 1 Department of Anthropology, University of California San Diego, 9500 Gilman Drive #0505, San Diego, CA, 92093, United States, 1 951-491-4161; 2 Global Health Policy and Data Institute, San Diego, CA, United States; 3 San Diego Supercomputer Center, San Diego, CA, United States; 4 University of California San Diego Medical Center, San Diego, CA, United States; 5 BitMind, Austin, TX, United States; 6 Herbert Wertheim School of Public Health and Human Longevity Science, University of California San Diego, San Diego, CA, United States; 7 Native Biodata Consortium, Eagle Butte, SD, United States; 8 School of Complex Adaptive Systems, Arizona State University, Tempe, AZ, United States

**Keywords:** blockchain, Indigenous Data Sovereignty, technology, proof of concept, Indigenous-led research collaboration

## Abstract

**Background:**

Historic and ongoing problematic practices regarding the collection, storage, and use of Indigenous health data have led to the need to ensure principles of Indigenous Data Sovereignty (IDS) are followed in research practices and technology development.

**Objective:**

This project, a partnership between UC San Diego and the Native BioData Consortium (NativeBio), sought to explore the practical application of blockchain technology and its potential to facilitate Indigenous-led research collaboration.

**Methods:**

This project first undertook purposeful relationship building with NativeBio to form a Community Advisory Board (CAB) for identifying community and technology needs for a blockchain research collaboration platform with an initial focus on genomic data. Over a 2-year project period, a series of public meetings and presentations at Indigenous-led conferences introduced the concept of exploring compatibility between blockchain and IDS principles, followed by iterative prototyping and co-design of a blockchain platform with NativeBio, using Ethereum as the underlying protocol.

**Results:**

Direct engagement with NativeBio and the CAB informed the initial design and development of a “b-IDS” proof-of-concept (POC) blockchain platform. The POC consists of three main components: (1) the web front-end layer, (2) the Ethereum network that executes the smart contract and blockchain storage aspects of the framework, and (3) the back-end database that stores off-chain interactions and data for future use with external genomic data repositories. After refinement of the POC, a community-based participatory research (CBPR) use case aligned with IDS principles was identified as a practical workflow and incorporated into the design of the POC for implementation.

**Conclusions:**

The findings from this project demonstrated the potential use of operationalizing IDS through blockchain technology with proactive and sustained engagement with Indigenous partners. Blockchain technology may have certain advantages over other data governance approaches and systems, facilitating timely oversight, shared decision-making and consent structures, and direct involvement of Indigenous communities in technology design, respecting the core principles of IDS and CBPR. Future development of the blockchain-IDS POC will need to incorporate other research practices and ethics frameworks to expand its use to other public health and biomedical research use cases.

## Introduction

Blockchain technology, which is generally synonymous with its most popular real-world application, Bitcoin, has been narrowly construed in the context of financial technologies (ie, “FinTech”) such as cryptocurrencies and nonfungible tokens. However, the application of distributed ledger technology, the backbone of blockchain applications, also has several commercial and business use cases, including in health care and social impact [1-5]. There are also efforts to ensure that blockchain technologies are operated for the betterment of modern society, such as the Blockchain for Good Alliance.

Critically, blockchain systems also interact with other technology layers, such as AI, federated learning approaches, and Internet of Things (IoT) and connected devices, which may interest American Indian and Alaska Native (AI/AN) communities and Indigenous scholars seeking innovative ways to address AI/AN health disparities and bridge the digital divide with distributed infrastructure in tribal communities [6]. In the context of biomedical research and health information exchange, blockchain has been positioned as a tool to promote the provenance and security of health data (eg, protected health information [PHI], consumer health information, whole genomes) shared between distributed parties, including biorepositories, academic medical centers, and commercial entities, but has rarely been reported for Indigenous use cases.

In the specific context of applying blockchain technology to health information and data associated with Indigenous communities, there has been little research or technology development that has been purposefully carried out with respect to Indigenous Data Sovereignty (IDS). IDS is defined as the authority of Indigenous entities to govern the collection, ownership, and application of their data, whether related to research or other purposes [7], and is grounded in the principles of Free, Prior, and Informed Consent (FPIC), as outlined in the United Nations Declaration on the Rights of Indigenous Peoples (UNDRIP). IDS is closely aligned with more Western research-centric efforts to encourage community-based participatory research (CBPR), which would encourage the inclusion of Indigenous communities in all parts of the research process [8]. Past national programs (such as the US National Institutes of Health’s All of Us Research Program) have been criticized for their storage of biospecimens from sovereign AI/AN communities, as well as no explicit representation from AI/AN researchers and tribal leaders on data access committees, highlighting ongoing and unaddressed concerns Indigenous communities have with the misuse of their PHI [9-11]. In response to these challenges, the nonprofit Native BioData Consortium (NativeBio) [9] was created as an independent data repository and research center, becoming the first Indigenous genomic biorepository led by Indigenous scholars and located on an AI/AN reservation.

As illustrated by the National Institutes of Health “All of Us” case study and the historic misuse of Indigenous data, the complex nature of tribal sovereignty and honoring IDS principles raises concerns about which governmental entities or partners have access to PHI managed by digital technologies, such as elected tribal leaders, public health agencies, researchers, government entities, and traditional knowledge holders. Inherently, specific blockchain technology features, if designed appropriately, can address some of these challenges by promoting user- and community-specific data privacy rules, such as through the use of automated smart contracts (ie, digital contracts stored on a blockchain that automatically execute when certain predefined conditions are met), dynamic consent structures (ie, procedures that allow research participants to review and renew their consent for use of their health information), and consensus mechanisms (ie, consensus mechanisms are the rules and procedures that ensure agreement in a decentralized blockchain network). This functionality can help to decentralize data governance in line with IDS [6].

Crucially, the potential use of blockchain to facilitate IDS and responsible data governance is rooted in the principle of full digital self-sovereignty, which is described as the ability for a collective of individuals (or groups of communities) to own, control, and manage their data throughout the data’s entire lifecycle in a distributed network, rather than relying on a central authority or external partner to make decisions without their direct involvement and input [12]. The modular design of blockchain-based technologies may also facilitate the incorporation of IDS principles by structuring data systems in a way that maximizes transparency and accountability for data use and sharing while minimizing the risk of unauthorized data access [1]. However, the realization of establishing compatibility between blockchain features, IDS principles, and real-world value for Indigenous communities is underexplored. In response, this paper presents the steps taken to develop—in collaboration with NativeBio—a proof-of-concept (POC) blockchain-based system (b-IDS) designed to effectuate sovereign data governance of AI/AN data, in alignment with the principles of IDS. The paper also reflects on lessons learned about the opportunities and limitations of such technologies for advancing IDS.

## Methods

### Overview

This study sought to explore the practical application of blockchain technology to the NativeBio context and how it could facilitate Indigenous-led research collaboration and responsible stewardship of data by operationalizing IDS in the underlying design of a technology system. To accomplish this, the project was carried out in engagement and consultation phases, followed by technology prototyping and iterative design that align with approaches in design science research (DSR) [13]. The first phase involved the development of and necessary relationship building with NativeBio and a separate Community Advisory Board (CAB) convened specifically for this project to advise on problem identification and motivation (DSR stage 1) and provide a wide range of perspectives on the proposed collaboration and technology design. The second phase included a series of public meetings and presentations to introduce the concept of blockchain and engage in listening sessions about opinions on its potential compatibility with IDS principles and define overall objectives of a blockchain solution (DSR stage 2; **Multimedia Appendix 1)**. A third phase involved key informant interviews with AI/AN scholars and tribal leaders exploring themes and shared opinions about digital technologies, blockchain, and IDS with these results published in a separate paper (related to DSR stage 2) [14]. The final phase involved prototyping of a blockchain platform (DSR stage 3—Design and Development) with different iterations that were evaluated, refined, and adjusted based on feedback from NativeBio and our CAB (DSR stages 4—Demonstration and 5—Evaluation) with results described in this manuscript (DSR stage 6—Communication).

### NativeBio and Community Advisory Board

In accordance with IDS principles, research team members from UC San Diego entered into a Memorandum of Understanding with NativeBio outlining the roles, responsibilities, rights, and expectations of researchers in relation to their collaboration with an Indigenous organization. In addition, the CAB was convened by NativeBio to advise on the design of the b-IDS prototype system and identify proper protocols for its operation and management. Prior cases of misuse of AI/AN data necessitated these approaches to ensure adequate oversight and input into the design of the proposed technology [15,16].

### Engagement With Indigenous Rightsholders

The need to develop reciprocal relationships with Indigenous communities and organizations, such as NativeBio and our CAB, is critical when conducting research and technology development with geographically and socioculturally diverse communities [17]. This approach created a space for NativeBio to voice concerns about technology during the earliest stages of design ideation, POC development, and initial user testing at a time when these systems are most amenable to change. It was important to frame the engagement process, including any co-design approach involving NativeBio, as strictly exploratory, meaning that we held no preconceived determinations about the design, choice, or purpose of the technology proposed to be developed, or even the form of management or governance of data on the blockchain-based system [18]. Iterative design processes centered AI/AN perspectives, while also allowing the freedom of exploration of a proposed technology in a dynamic cultural, political, and regulatory environment. The research team also organized 3 in-person meetings and events, which informed the initial development of the blockchain POC with the CAB.

### Blockchain POC Development

The first decision in blockchain POC development is to assess whether blockchain technology is actually needed, while also substantiating its need in comparison to other traditional health informatics and digital health systems. Building on work first published by Wüst and Gervais [19], we use the framework developed by Li et al [20] to assess the suitability of blockchain technology for sharing health care data, and specifically adopted for Indigenous data (see Table 1 for criteria in decision-making). Based on the assessment of these decision-making criteria, blockchain was deemed well-suited for the operationalization of IDS, including in comparison to trusted third-party frameworks and also in the selection of a public permissioned blockchain architecture under a consortium model (explained in more detail in a future section).

Another important consideration is how data interacting with a blockchain is managed, particularly in health-related applications. Specifically, data managed by a blockchain can either be “on-chain” (data stored with individuals contributing health data) or “off-chain” (data stored in a biorepository or biobank), depending on the preferences of the community [5,21]. Generally, there is a preference for “off-chain” storage due to cost-efficiency, limited technology infrastructure on tribal lands, and the request for handling AI/AN data by an AI/AN-managed entity [22,23]. With the “off-chain” model, it is possible for a limited degree of data (eg, metadata or state of data) to be kept on the blockchain to allow researchers and other parties to query the blockchain for the availability of certain data attributes (as permitted by the governing entity of the blockchain) without the ability to access individual-specific data [23].

With these fundamental blockchain architecture principles assessed and determined—specifically adopting a public permissioned blockchain consortium model and using both on-chain and off-chain data management approaches—we proceeded with developing the POC and exploring practical use cases aligned with IDS and NativeBio real-world needs.

**Table 1.** Blockchain technology for sharing health care data framework (Li et al [20]).

Criteria for blockchain use	Yes or no and rationale	Applicable blockchain architecture
Step 1 Do you need to share data?	Yes—Sharing of certain types of data is critical for making decisions on research associated with Indigenous data if done so in alignment with IDS^a^ principles.	Data storage approaches (on-chain vs off-chain) Decision-making and consensus mechanisms on sharing data Smart contract execution of access and data sharing
Step 2 Can you share via trusted third parties?	No—Historical mistrust in storage and control of Indigenous data by government agencies and other third parties makes it hard to justify centralized systems.	Consortium-based models for shared governance and retaining data ownership and control Use of decentralized autonomous organization (DAO) for decision-making
Step 3 Are data integrity and immutability critical?	Yes—Accuracy, traceability, auditability, and integrity of data sharing and decision-making are required.	Use of distributed ledger technology Transparency in decision-making through immutability and cryptographic hashing
Step 7 Is transparency toward actors important?	Yes—Fostering trust among Indigenous organizations and partners and those who want to use Indigenous data in alignment with Indigenous people’s needs and IDS principles is central to the system.	Use of a public permissioned or permissionless blockchain to build trust and accountability in data management Balancing this approach with privacy-preserving elements
Steps 5‐9: #5: Are the identities of actors known? #6: Are all actors trusted? #7: Do you need to involve patients? #8: Is cost a limiting factor? #9: Are the identities of actors known	#5 Yes—Verifying all identities is essential, need to support interoperability and authenticated interactions across users. #6 No—Trust is not on a single actor but instead a distributed network governed by IDS principles. #7 N/A—Participants on our blockchain do not fit the strict definition of patients. #8 Yes—Cost is a consideration for Indigenous technology infrastructure. #9 Yes—Public verification while controlling access and maintaining levels of privacy and security is optimal.	Use of a public permissioned consortium blockchain architecture Incorporation of IDS principles into smart contract execution

a IDS: Indigenous Data Sovereignty.

### Ethical Considerations

The qualitative portion of this study that involved human participants, with results reported in a separate published paper, was reviewed and deemed exempt by the University of California, San Diego Human Research Protection Program Institutional Review Board (IRB #807171) [14]. Human participants were compensated for their participation in a 60-minute interview. No other part of the study involved human participants research.

## Results

### Overview

**Figure 1.**
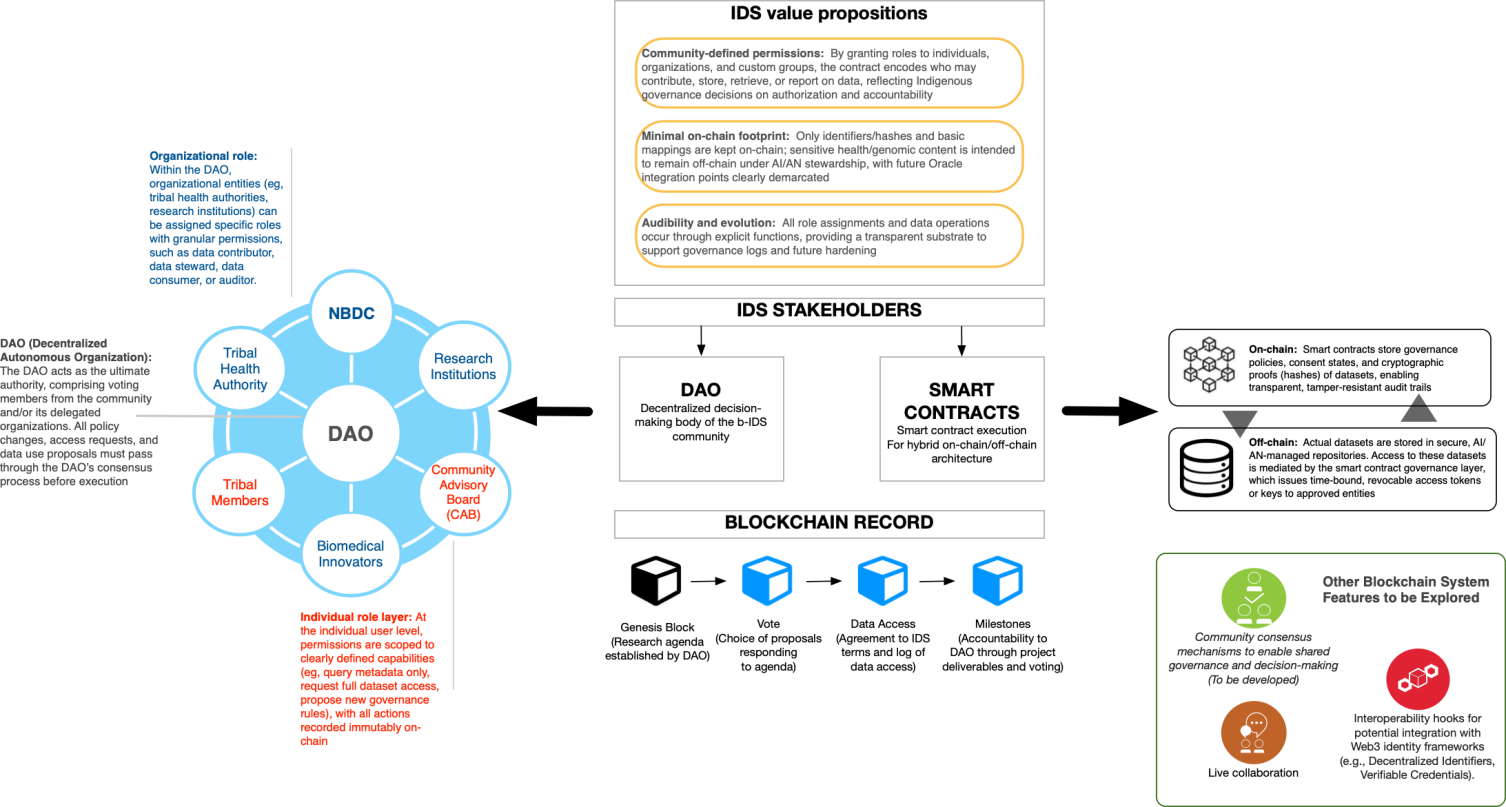
Visual depiction of b-IDS blockchain system architecture. The figure illustrates the hierarchical structure of the b-IDS blockchain architecture, starting with value propositions centered on IDS, self-governance, and distributed and trusted biomedical innovation. Rightsholders, including tribal health authorities, research institutions, and community members, are mapped directly to DAO members for consensus-driven decision-making. At the same level, smart contracts execute governance rules and transactions, writing immutable records to the blockchain for transparency and auditability, and handling rules-based access control of sensitive and private data. Complementing this on-chain core, an off-chain database on the side handles scalable storage of sensitive data, ensuring hybrid efficiency while aligning with IDS principles. AI/AN: American Indian and Alaska Native; DAO: decentralized autonomous organization; IDS: Indigenous Data Sovereignty; NBDC: NativeBio Data Consortium.

Following the initial engagement phases with NativeBio and the conceptualization of the b-IDS blockchain framework, the study team selected Ethereum as the underlying blockchain protocol for the b-IDS POC. Ethereum was chosen for its mature smart contract environment, robust decentralized governance capabilities, ability to integrate with both on-chain and off-chain data architectures, and robust tooling around decentralized autonomous organizations (DAOs) and the open-source developer ecosystem. A core design objective was to enable digital self-sovereignty by embedding IDS principles directly into the system’s governance and access control logic as a “public permissioned” blockchain. To achieve this, we developed a flexible governance framework centered on DAO mechanics. In this model, Indigenous community members or their designated representatives retain explicit, programmatically enforced authority over any use of community-provided data or related decision-making (see Figure 1 for a visual depiction of the design).

This governance system was structured around multilayered permissions and role hierarchies in Ethereum smart contracts. The permissioning model supported both positive control (explicitly granting access to approved parties) and negative control (revoking or suspending access rights) in real time through smart contract updates designed to reflect Indigenous governance norms. This ensures that any change in consent or governance status is immediately enforceable across the system. To safeguard potential use and access to sensitive health and genomic data while maintaining verifiable governance, the POC also implements a hybrid on-chain or off-chain architecture. The DAO framework was further extended to allow modular governance logic, enabling communities to tailor decision-making rules, such as quorum requirements, voting thresholds, or delegated authority structures, to their own cultural, legal, and political contexts. As Tribal citizens are both Tribal and American citizens, the ability to hybridize individual autonomy with collective decision-making under the rubric of IDS where smart contracts can be conditioned upon adherence to relevant tribal statutes may create ideal conditions for the protection and use of genetic data for the betterment of AI/AN health. Further, modularity ensures the governance and permission-based system can be specific to individual communities while still running on a shared Ethereum-based infrastructure. Rapid prototyping followed an iterative build-test-refine cycle, with each iteration evaluated by NativeBio and the CAB, allowing feedback and informed refinements ranging from the user interface and governance system to decision-making workflows aligned with the mission of NativeBio to advance AI/AN self-governance.

### b-IDS Blockchain Technical Framework

The b-IDS POC was deployed on a public Ethereum blockchain to leverage its mature developer ecosystem, security guarantees, and interoperability with the wider Web3 infrastructure. While the underlying chain is public and globally verifiable, the governance framework is permissioned through the b-IDS DAO, ensuring that only authorized participants can propose, vote on, or execute platform governance actions. The POC consists of three main components: (1) the web front-end layer, (2) the Ethereum network which executes the smart contract and blockchain storage aspects of the framework, and (3) the back-end database that stores off-chain interactions and data for future integration with the NativeBio biological resources repository. For the first layer, we focused on developing an easy-to-use and straightforward user interface/user experience for distributed decision-making and governance for Indigenous research collaboration. This integrated front-end layer communicates directly with the framework’s Ethereum Network b-IDS DAO and the smart contract layer for the execution of specific research collaboration processes.

Permission structures in the b-IDS POC are governed through integration with widely available crypto wallets (eg, Coinbase Wallet, MetaMask), which authenticate users, verify DAO membership, and mediate access to permissioned governance and data-sharing functions. While the underlying blockchain operates on Ethereum’s Proof-of-Stake (PoS) consensus, governance of the b-IDS platform itself is conducted through the permissioned DAO. Permission structures governed by the integration of readily publicly available crypto wallets (eg, Coinbase mobile app) mediate access to the DAO and permissioned-based decision-making and data access. For consensus, the b-IDS POC used a proof-of-authority consensus mechanism. All platform-level decisions are recorded on the public Ethereum network and tied to member identities, with execution governed by DAO-defined voting rules (eg, simple majority, weighted voting by role). DAO participants are approved by the community and can have their governance privileges revoked in cases of unethical behavior, which is a process strengthened by the transparency of blockchain records within a predefined community network not accessible to external parties.

The b-IDS Ethereum Network also included 2 key application layers: a platform-specific ERC-20 token for the quantification and possible tokenization of platform decision-making and participation and a suite of smart contracts to execute research management-specific workflows and terms specific to IDS principles (eg, adherence to tribal IRB requirements, proof of data attribution for potential benefits sharing). Shared governance of the platform is conducted via the DAO and was initially composed of the management team of NativeBio but can be populated with a set of broader Indigenous rightsholders during future testing and implementation (eg, CAB members, tribal elders, Indigenous community members, vetted researchers). Membership of participants for b-IDS can be validated by the DAO and participating community member nodes in the decentralized community based on needed expertise, credentials, and possible tribal affiliation. DAO participants can also be further subgrouped into different Indigenous communities (eg, tribal nation, elders, administrators, external experts, contractors) or expertise areas. DAO participants act as the “Authority” nodes that oversee the research collaboration processes and permissions written to the blockchain following community consensus and review by the appropriate tribal governing bodies.

### Application of Use Cases

Upon the completion of prototyping of the b-IDS platform with different iterations of the POC evaluated, refined, and adjusted based on user feedback, a clear CBPR use case aligned with the principles of IDS was developed based on feedback from NativeBio and the CAB. Each phase and within this CBPR use case represents a new transaction event that creates a timestamped cryptographically hashed “block” of data that records decision-making as outlined below (see Table 2 for a detailed description of the workflow for the community-initiated use case).

**Table 2.** Workflow for community-initiated use case.

Phase	Description	Blockchain action	Example specific to NATIVEBIO^a^
Phase I: DAO^b^ Research Initiation and Broadcast	A research agenda is defined by the DAO and broadcast to vetted researchers, academic institutions, or other partners approved to be in the b-IDS network who may be able to carry out a proposed project that aligns with the solicitation. Within this phase, being able to propose a project that is responsive to community needs is the primary focus.	Research agenda approved by DAO in alignment with community needs Community members participating in DAO can vote and approve research areas or topics before they are broadcasted Smart contracts execute and store decision-making information about voting history, research agenda details, and updates to data or other DAO actions (eg, research responses) through a consensus algorithm.	Similar to a Request for Proposals, the b-IDS DAO agrees to propose a set of research topics that will use genomic data in the NativeBio biological resources repository. Community members of the DAO help vote for project areas that will directly benefit their community and choose a topic.
Phase II: Researcher Response and Network Voting	After the DAO has broadcasted research topics or a request for proposals within a specific topic area to the b-IDS network, researchers prepare and respond with their submissions, or scope of work directly on the b-IDS system, detailing their approach to address the priority needs.	Researchers with vetted credentials as members of the b-IDS network (nonvoting and not part of DAO) submit research proposal for community voting Community members and DAO vote on proposals with the greatest potential scientific and community impact Smart contract execution of research proposals, voting history, and access to data or DAO actions.	Researchers that have been vetted by the DAO are able to submit proposals directly on b-IDS but need to do so with clear consideration of community benefit. Community members with voting privileges and the DAO then vote to determine which proposals submitted will be approved. This inherently requires researchers to clearly communicate both scientific merit and community benefit in their proposals.
Phase III: Research Execution Phase	The third phase begins the process of researchers carrying out the aims proposed and approved by b-IDS. First, when a research proposal is accepted, a smart contract is executed between the DAO and researcher recording the type and terms of the research and researcher responsibilities as well as the terms of token compensation.	Smart contract on the blockchain executes agreement to gain access to genomic data stored off-chain and other terms researcher is required to agree to in alignment with IDS principles (eg, tribal IRB^c^ requirements, data use, data return requirements) b-IDS records the approval of access to data and agreement to IDS^d^ principles and broadcasts to network Access logs that are secured by the smart contract and DAO are recorded to ensure a full history of data access to ensure full auditability and optimize security while ensuring the privacy of sensitive and private data.	Researchers A, B, and C are chosen by the community and the DAO to carry out research projects using NativeBio genomic data. Their access to data and any research support is self-executed through smart contracts that clearly and transparently indicate agreement to IDS principles. The process of award and agreement is transparent, immutable, and broadcast to the b-IDS network. Once smart contract execution is complete, researchers begin their projects.
Phase IV: Research Milestones	This phase ensures that ongoing research continues to align with the values, priorities, and evolving needs of the community. Milestones are communicated transparently through regular reporting and translation of progress to b-IDS that can then be voted on by community members and the DAO for continued data access and support. Community members and the DAO can also express concern or other feedback based on preliminary results. This process is repeated until the project closes (or is closed), where final results are shared, translated, and disseminated to the community.	b-IDS network reviews the research updates and votes through a decentralized process to determine if the research should continue. Voting can be delegated or restricted to certain nodes. Once a majority vote with a minimum threshold is achieved, the researcher may proceed with the next phase of the proposal, until the next milestone is reached. Smart contract can execute requirements for return or validating proper disposal of data once milestones are complete.	Researcher A provides progress reports on milestones achieved for their project, community votes to approve and some community nodes and DAO members provide feedback. Researcher B fails to provide relevant progress on milestones. Community members and DAO vote to not continue research project, and smart contract is executed for close-out requirements. Researcher C provides relevant progress on milestones, and community members and DAO vote to not continue research as clear community benefit is not shown or described.
Blockchain record	Upon completion of a research project, there is a complete and immutable history of all decisions, transactions, data access, and outcomes associated with the project. This ensures better accountability, auditability, and responsiveness to community and DAO needs.	—^e^	—

a NATIVEBIO: Native BioData Consortium.

b DAO: decentralized autonomous organization.

c IRB: institutional review board.

d IDS: Indigenous Data Sovereignty.

e Not applicable.

Within this CBPR workflow, the primary outcome is researchers conducting genomic studies that align with community priorities while maintaining transparency and accountability through recurrent updates and milestone reports that are published to and voted for on the blockchain platform. Researchers, academics, or institutions (ie, researcher nodes) must first be approved nodes who can participate in the b-IDS network. A prevetting process can either be done on-chain (by broadcasting and having the DAO vote on if a researcher is to be given permissions as researcher nodes) or done off-chain in which the DAO determines who to include as trusted members on the network. These researcher nodes will need to adhere to IDS principles in order to participate, with agreement through smart contract execution and memoranda of understanding.

A researcher’s proposal and subsequent progress on their project can only advance when they receive a majority vote, or community consensus, at each phase of the community-mediated management process that is recorded in an immutable fashion on the b-IDS system. This workflow directly benefits the community as they can become actively involved in decisions about research that involves their individual or community-based data, and researchers are accountable to these communities through frequent disclosure and dissemination of their research back to community members in accessible formats. The primary benefit to researchers is potential support and access to genomic data to conduct work directly tied to identified AI/AN community needs, rather than passive inclusion of AI/AN communities in a larger project involving other demographics. Though not part of the DAO, any community member who is approved on the b-IDS as a node can be given appropriate voting rights for specific proposals that involve use of their data and can also delegate their voting authority to other nodes (eg, tribal leaders, tribal health organizations), if desired.

At the completion of a research project’s lifecycle on b-IDS, a full and complete immutable record of all actions and shared decisions made (ie, data provenance), including proof of consensus of community and DAO approval for all stages of the research process (eg, approval of research topics, proposals, milestones, and any final deliverables), is recorded and can be broadcast to the distributed b-IDS network. Future design can also incorporate tokens that can be allocated to nodes to facilitate decisions, transactions, and incentives on b-IDS based on further testing and ownership distribution aligned with IDS. Evidence of community input in the scientific process is also achieved and can translate to potential coauthorship in scientific dissemination or future benefit-sharing based on any terms agreed upon in the original research proposal phase (eg, IP ownership, technology transfer, irrevocable license to use the benefits of discoveries).

## Discussion

### Principal Findings

This study explored the application of blockchain technology to Indigenous health research and its compatibility with IDS through the development of the b-IDS platform with NativeBio. The main findings were the identification of a relevant use case for blockchain by focusing on the development of a CBPR-focused workflow and governance process for shared decision-making regarding research uses of Indigenous data. In a previous published paper, we demonstrated the early conceptualization of a blockchain technology approach to safeguard Indigenous genomic data and its overall technical framework [23]. For this study, we advanced this conceptualization and purposefully worked with NativeBio and our CAB to identify specific blockchain technology features and platform design considerations, as well as implement a specific data use case to practically embed IDS into a broader data and research management workflow.

Beyond the prototyping of b-IDS, we also believe that the study contributes to establishing a better understanding of how to work with Indigenous communities when evaluating and designing technology. Specifically, we believe the study contributes the following key takeaways: (1) before technology choice or design can even begin, purposeful time, listening, and relationship building with Indigenous communities are not suggested, but required; (2) determining a use case for your technology development may change based on an Indigenous community’s needs, so Indigenous allies should have no preconditions to their technology engagement or approach communities with time-sensitive funding opportunities and grant deadlines absent an existing relationship; and (3) principles of IDS need to be built into the technology architecture itself, which required the research team to purposefully map blockchain design elements to IDS with community partners.

Though the overall objective of this project was to address long-standing poor data governance and management of genomic data issues commonly experienced by Indigenous communities whose data are becoming increasingly sought by researchers and institutions without regard to IDS, actual implementation and operationalization of IDS principles into new and novel technology will require further reflection and partnership that will likely take a generation to realize. Acknowledging this, before moving forward with the implementation of b-IDS to reach community consensus on specific research projects that could access Indigenous genomic data in the NativeBio data repository, we instead have focused on using the platform to reach internal consensus among DAO members (which include NativeBio and CAB members) about what research agenda items need prioritization to bring to funders, community organizations, and other actors that may want to participate in the consortium model. This also brings direct benefit to our Indigenous partner NativeBio, as it presents an alternative model to organizational governance, where decisions about what research areas NativeBio prioritizes are more transparent and accountable to the communities it serves.

What we also learned from this process of engagement and technology development with NativeBio is that the compatibility of blockchain systems or any other digital health or data governance technology depends on how well it reflects the social, cultural, and technological needs of distinct AI/AN and Indigenous communities [24]. In practical terms, this means that specific blockchain features, such as smart contracts, secure cryptographic signing, dynamic consent, immutable records, permissioned platforms, consensus mechanisms, Web 3.0 features, and off-chain storage, must be designed in alignment with Indigenous organizational and community needs if they are to uphold IDS principles and have practical value [23]. Although other studies have examined blockchain’s application for research governance (eg, managing the academic publishing process, peer-review, promoting open science, tokenizing academic contributions) and management of genomic data (eg, in the context of self-sovereign identity, decentralized storage, ontologies, consent processes, and even use of nonfungible tokens), ours is the first to our knowledge to explicitly address blockchain prototyping in the context of IDS [25-31].

We also learned that blockchain-based systems must also provide degrees of privacy and control to individuals as well as to broader communities or “delegates” representing those users. This requires additional privacy-preserving protocols and decentralized management of sensitive data, such as the off-chain storage approach described in our POC [5,32]. Additionally, there needs to be a focus on designing data governance systems that are sustainable and earth-friendly, meaning that the implementation of b-IDS will need to adopt the use of energy-efficient technologies, such as the PoS consensus mechanism used in our POC (eg, an article by Platt et al [33] substantiated that the energy consumption of PoS is vastly lower than Proof-of-Work, which is the consensus mechanism used for Bitcoin transactions), while also using infrastructure that prioritizes low power consumption and reduces any environmental impact (eg, renewable powered microdata centers) [33-36].

It is essential to recognize that engagement will vary across different Indigenous contexts. Willingness to adopt next-generation data systems also depends on local governance, cultural priorities, and IT infrastructure [7]. Variability is not a limitation but a reflection of sovereignty itself, underscoring the need for community-by-community consultation and consent before developing “private” or “permissioned” blockchain systems. Hence, though our original inception of b-IDS was to create a potential alternative to federal biorepositories with and as NativeBio, the consultation and design process ultimately generated broader sociopolitical discussions about Indigenous authority over health and research data. Because AI/AN data are often governed by tribal laws that exceed federal protections, blockchain systems will only be viable if IDS principles are built into their architecture, something we attempted to achieve through the use of smart contracts that embed IDS principles into their operation [7].

NativeBio has also endeavored to follow the Canadian First Nations’ Indigenous Governance Centre trademarked principles called OCAP (Ownership, Control, Access, and Possession) in their work as the strongest assertion of IDS. Relevant to this pursuit, First Nations, Inuit, and Métis communities have felt that OCAP has been ignored by settler governments, which led to the Assembly of First Nations of Canada approving a nationwide resolution from 634 Indigenous Nations demanding that Canada, in partnership with public and private funders, cease and desist the collection of Indigenous DNA within their territories, Indigenous knowledge systems, or through incidental collections such as sewer water and related environmental waste systems until they have legally binding agreements for service and benefits derived from the use of their PHI [37].

Future work on blockchain systems, such as b-IDS, should crucially address how genomic data can generate direct benefits for Indigenous communities and who regards these data as relational and should be the primary beneficiaries of their use in scientific discovery [38,39]. A critical gap in current governance tools is the absence of mechanisms to appropriately attribute contributions and ensure benefit-sharing when communities’ data are used by academic institutions or biotechnology companies. Blockchain systems, with their ability to tokenize data and record immutable transfers of value, offer one potential pathway to formalize such attribution and create enforceable benefit structures. Properly designed, these approaches could allow communities to pool data for shared health and social goals while safeguarding sovereignty.

Finally, at its foundation, IDS is grounded in the principle of FPIC, as outlined in the UNDRIP. FPIC affirms the authority of Indigenous communities to give or withhold consent regarding data collection, access, and use, ensuring that all data-related activities uphold their sovereignty. In addition to FPIC, the CARE (Collective Benefit, Authority to Control, Responsibility, and Ethics) principles ensure that data management serves the collective interests of Indigenous communities and prioritizes ethical and culturally aligned practices in all stages of data governance (eg, data collection, use, and management) [24,40]. Future development of our b-IDS POC should further incorporate diverse research practices and ethics frameworks such as FPIC, CARE, and those specific to digital health and IDS, such as the work described in Cordes et al [24] to expand to other public health and biomedical research use cases. This may help a broader set of researchers, data holders, and community groups engage in technology-mediated CBPR with interested communities.

### Limitations

Although we strived to develop and prototype a blockchain-based system rooted in IDS principles that meets the research and data governance needs of Indigenous people, the b-IDS system has inherent limitations that need to be addressed in future studies in order to assess its viability for community adoption and technology implementation. For example, though our proposed blockchain-mediated workflow trusts researchers to accurately report progress and milestones for community voting and consensus, it does not include a process to independently verify real-world inputs or impact. This may especially be challenging when information asymmetry limits the ability for community members to assess the impact of research or its accountability to community goals. In response, integration of additional CBPR-based validation mechanisms (eg, community audits) could ensure better research accountability. Although community consultation and subsequent prototyping of b-IDS identified the need for privacy-preserving protocols and decentralized data management, particularly in the context of sensitive genomic data, our proposed hybrid on-chain and off-chain architecture has inherent limitations. Chief among these is the need to establish a balance between transparency and trust across a distributed network and use of data for research purposes. For example, limiting consortium and node visibility to deidentified metadata or smart contract execution logs may protect the privacy of genomic-related data but still inadvertently exposes sensitive community activities that need to be further explored in use case–dependent implementation work. Finally, and most crucially, more thoughtful input with a broader set of Indigenous community users/data holders, more inclusive user validation testing, and further assessment of real-world value to Indigenous communities will be needed in order to assess the viability of actual technology adoption and implementation.

### Conclusions

Advancing IDS is not only a matter of ethics but also a matter of public health: without sovereign governance tools, mistrust in data systems will continue to undermine public health and digital technology development involving Indigenous communities. Further, respect for sovereignty also requires allowing for refusal, modification, or alternative pathways, rather than expecting uniform uptake of technology. This is not a barrier but rather a fundamental expression of sovereignty itself, and it needs to be respected in any technology and data governance design. Hence, the contribution of our b-IDS prototype lies less in blockchain itself than in demonstrating how digital architectures can be designed to uphold sovereignty and strengthen Indigenous health governance while also supporting autonomy, transparency, and eventual data sharing to advance Indigenous and broader global health outcomes.

## Supplementary material

10.2196/90247 Multimedia Appendix 1 Additional information on listening sessions with Indigenous groups. 1, 2

## Abbreviations

AI/AN: American Indian and Alaska Native
CAB: Community Advisory Board
CARE: Collective Benefit, Authority to Control, Responsibility, and Ethics
CBPR: community-based participatory research
DAO: decentralized autonomous organization
DSR: design science research
FPIC: Free, Prior, and Informed Consent
IDS: Indigenous Data Sovereignty
IoT: Internet of Things
NativeBio: Native BioData Consortium
OCAP: Ownership, Control, Access, and Possession
PHI: protected health information
POC: proof-of-concept
PoS: Proof-of-Stake
UNDRIP: United Nations Declaration on the Rights of Indigenous Peoples
