# Supplementary material for: Development of a Blockchain-Based Platform to Enable Indigenous Data Sovereignty and Shared Research Participation With Indigenous Communities: Technology Prototyping and Community Engagement Study
Source: J Med Internet Res. 2026 Jul 28;28:e90247. doi: 10.2196/90247 (PMC13412011; doi:10.2196/90247)
Supplement: Multimedia Appendix 1 [file jmir-v28-e90247-s001.docx]

**Multimedia Appendix**

**Additional Information on Listening Sessions with Indigenous Groups**

In addition to our direct engagement with NativeBio and our CAB, two domestic (*Indigenous Innovation, Data Economies, and AI (IDEAS) Summit* in 2023 in Phoenix, AZ and *U.S. Indigenous Data Sovereignty Network Conference (USIDSN)* in 2024 in Tucson, AZ) and one international event (special session at the *World Federation of Public Health Association (WFPHA) Congress on Public Health (WCPH)* co-hosted by the WFPHA Indigenous Working Group and Global Health Equity and Digital Technology Working Group in 2023 in Rome, Italy) informed the initial development and helped refined and conceptualize a POC with the NBDC and CAB. The general discussion points of the events focused on: (1) describing how digital health technologies, such as blockchain, can operationalize IDS; and (2) creating an open space for Indigenous futurists to co-ideate on ethical, legal, and social implications (ELSI) relevant to the design of health technologies handling genomic and other sensitive health data from Indigenous Peoples.

There was diversity in discussion topics, but some of the major themes from these engagements included discussions regarding the need to specifically address AI/AN privacy concerns and the role of technology in managing genomic data and accessing personal health information. General observations from these public events (which were not recorded or used for qualitative analysis) were presented back to the NBDC and CAB, which then guided initial conversations as to how b-IDS should function. Specifically, there was an emphasis from the CAB that the proposed b-IDS system should not manage or have direct access to an individual’s genomic data and should simply act as platform that records whether decisions were made by a community-authorized authority. This informed our design focus on a decentralized blockchain system that can act as a facilitator of responsible data governance and has safeguards against external parties breaching data confidentiality.

From a technical standpoint, this approach is supported by permissioned blockchain systems, which can allow for off-chain storage of actual participant genomic data and also offer varying levels of data management and access for any other information or data being shared (e.g., participant metadata such as age, geographic location, select biomarkers)^11^ An additional important consideration that was frequently discussed at these events was the importance of earth-friendly computation methods and the environmental impact of blockchain use that were then built into a minimal footprint design. Other key themes were mapped to the Consolidated Framework for Implementation Research – Expert Recommendations for Implementation Change (CFIR-ERIC) Matching Tool as part of a parallel effort using implementation science to understand how best to design a system that prioritizes the responsible use of Indigenous data (see **Table 1**).

List of key themes that were mapped to the Consolidated Framework for Implementation Research – Expert Recommendations for Implementation Change (CFIR-ERIC) Matching Tool as part of a parallel effort using implementation science to understand how best to design a system that prioritizes the responsible use of Indigenous data:

**Table 1. Event IDS Thematic Output(s) Matched with CFIR-ERIC Topic(s).**

| **Event** | **Thematic Output(s)** | **CFIR-ERIC Topic(s) (Perceived Barriers)** | **Implementation Level** | **Descriptions for Perceived CFIR-ERIC Barriers** |
| --- | --- | --- | --- | --- |
| IDEAS (2023) | Adaptation | - Adaptability | Intervention Characteristics | Tribal authorities do not believe that the innovation can be sufficiently adapted, tailored, or re-invented to meet local needs. |
|  | Cost | - Cost | Intervention Characteristics | Tribal entities believe the innovation costs and/or the costs to implement (including investment, supply, and opportunity costs) are too high. |
|  | Operationalization of IDS | - Culture | Inner Setting | Cultural norms, values, and basic assumptions of the community hinder or promote implementation. |
|  | Environmental Impact | - Cost (Proposed) | Outer Setting (Proposed) | Working Definition: The expected and unexpected, environmental costs to design and implement a digital health system or technology. |
| WCPH (2023) | Incentivization | - Organizational Incentives & Rewards | Inner Setting | There are no tangible (e.g., goal-sharing awards, performance reviews, promotions, salary raises) or less tangible (e.g., increased stature or respect) incentives in place for individuals to provide data. |
|  | Relative Importance | - Relative Priority | Inner Setting | Community leaders perceive that implementation of the innovation is not a priority relative to other initiatives or activities. |
| USIDSN (2024) | Supporting Parties and Regional Pilots | - Champions - External Change Agents | Process | Individuals or traditional knowledge holders acting as champions who ‘drive implementation in a way that helps to overcome indifference or resistance by key stakeholders are not involved or supportive.  Individuals, funders, and policymakers from an outside entity formally facilitating decisions to help move implementation forward are not involved or supportive. |
|  | Elder Engagement | - Opinion Leaders | Process | Involvement of opinion leaders, including individuals, groups, and elders who have formal or informal influence on the attitudes and beliefs of their colleagues with respect to implementing the intervention. |
